# The Charlson Comorbidity Index Predicts Clinically Relevant Postoperative Pancreatic Fistula in Patients Undergoing Distal Pancreatectomy Not Pancreaticoduodenectomy

**DOI:** 10.1002/wjs.12557

**Published:** 2025-03-24

**Authors:** Hiroki Imamura, Yoshito Tomimaru, Shogo Kobayashi, Kazuki Sasaki, Shinichiro Hasegawa, Daisaku Yamada, Hirofumi Akita, Takehiro Noda, Hidenori Takahashi, Yuichiro Doki, Hidetoshi Eguchi

**Affiliations:** ^1^ Department of Gastroenterological Surgery Graduate School of Medicine Osaka University Osaka Japan

**Keywords:** Charlson comorbidity index, distal pancreatectomy, postoperative pancreatic fistula

## Abstract

**Background:**

Postoperative pancreatic fistula (POPF) is a severe complication after pancreatectomy. The preoperative prediction of POPF would benefit patients by providing postoperative management tailored to each patient based on the risk for POPF. The Charlson Comorbidity Index (CCI), which assesses the severity of patient comorbidities, has been associated with postoperative complications in various surgeries. However, its relationship with POPF remains unclear. This study investigates the impact of CCI on the development of POPF.

**Methods:**

This retrospective study reviewed 597 patients who underwent pancreatectomy from 2010 to 2020, of whom 219 underwent distal pancreatectomy (DP) and 378 underwent pancreaticoduodenectomy (PD). Significant factors were assessed in association with clinically relevant POPF (CR‐POPF) using a logistic regression model. K‐means clustering was employed based on the body mass index, pancreatic thickness, and CCI score to stratify patients by the risk for CR‐POPF.

**Results:**

Higher CCI scores were significantly associated with an increased incidence of CR‐POPF, particularly in patients undergoing DP, whereas such association was not observed in patients undergoing PD. Multivariate analysis identified male sex, BMI > 25.95 kg/m^2^, pancreatic thickness > 9.01 mm, and CCI score > 4 as independent predictors of CR‐POPF in the DP group. A predictive model incorporating these factors demonstrated moderate accuracy (AUC = 0.6750) in stratifying patients into high‐ and low‐risk groups for CR‐POPF.

**Conclusion:**

CCI is a significant predictor of CR‐POPF, especially in patients undergoing DP. By integrating CCI with other factors, it was feasible to develop a predictive model with high diagnostic accuracy.

## Graphical abstract

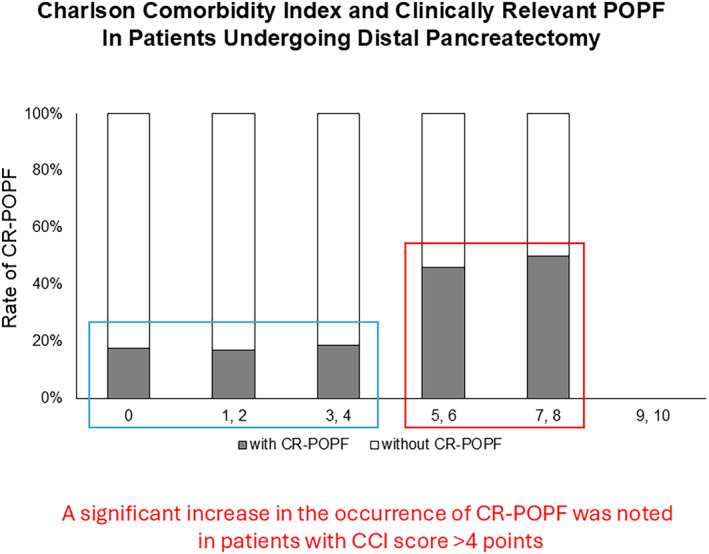

## 1 Introduction

Postoperative pancreatic fistula (POPF) is one of the most common life‐threatening complications after pancreatectomy, with 2.5%–5.6% of mortality rate [1]. Preoperative assessment of the risk of POPF is essential since postoperative management should be tailored to each patient according to the risk for POPF. That is, patients at high‐risk for POPF may benefit from preoperative nutritional support [2] or postoperative use of somatostatin analogs [3], whereas low‐risk patients may benefit from more aggressive postoperative management such as the early drain removal strategy [4].

Risk factors for POPF have been extensively investigated, among which the pancreatic status, such as texture or thickness [5], is well known as significant contributors to POPF. In contrast, although some comorbidities, such as histology of pancreatic tumor [6], diabetes mellitus [7], or renal failure [8], have also been reported to be associated with POPF, their relevance to POPF has not been as clearly revealed as the pancreatic status. Therefore, future studies are expected to reveal the impact of patients' comorbidities on the formation of POPF.

Charlson Comorbidity Index (CCI) was originally reported to evaluate the prognosis of patients by classifying the severity of their comorbidities [9]. It is also associated with postoperative prognosis in patients undergoing surgical resection including pancreatectomy [10, 11]. Recently, some articles described the association of CCI with postoperative complications [12, 13], among which Smith et al. reported the significant association between CCI and postpancreatectomy complications [14]. However, their study exclusively aimed at patients with pancreatic neuroendocrine tumors, and POPF composed of only 0.6% of the complications, probably due to the obscure definition of POPF. Therefore, further studies are warranted to evaluate the relation between CCI and the occurrence of POPF.

In this context, in this study, we have utilized CCI as an objective score to assess the comorbidity of patients and investigated its impact on POPF.

## 2 Materials and Methods

1. Patients

**FIGURE 1:**
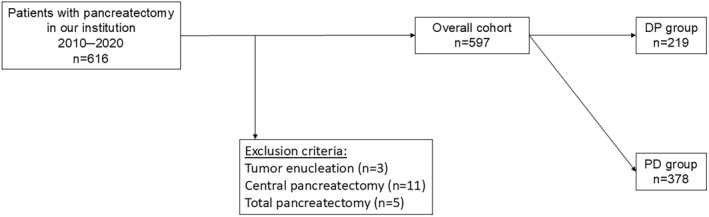
The diagram of patient selection. Consecutive 616 patients who underwent pancreatectomy at our institution from 2010 to 2020 were retrospectively reviewed. Of these patients, 19 patients were excluded because 3 underwent tumor enucleation; 11 underwent central pancreatectomy; and 5 underwent total pancreatectomy (including repeated pancreatectomy). The remaining 597 patients were evaluated for analysis and divided into the DP group and the PD group.

The current study was performed in compliance with the Declaration of Helsinki and was approved by the ethical committee of our hospital (approval number 24079). Consecutive 616 patients who underwent pancreatectomy at our institution from 2010 to 2020 were retrospectively reviewed (Figure 1). Of these patients, 19 patients were excluded because 3 underwent tumor enucleation; 11 underwent central pancreatectomy; and 5 underwent total pancreatectomy (including repeated pancreatectomy). Of the remaining 597 patients, 219 patients underwent distal pancreatectomy (DP) and 378 patients underwent pancreaticoduodenectomy (PD). Patient characteristics were reviewed for sex, age, BMI, neutrophil‐lymphocyte ratio (NLR), pancreatic thickness, the use or nonuse of neoadjuvant therapy, histology of pancreatic tumor, operative approach, type of procedure, operative time, blood loss, intraoperative transfusion, and the occurrence of clinically relevant POPF (CR‐POPF). Every patient was screened for his/her comorbidities at the initial visit using CCI in accordance with the previous report [9]. POPF was graded based on the definition of ISGPS [15]. The data of patients undergoing DP at our institution from 2021 to 2023 were also collected as a validation cohort.

2 Surgical Procedure

In DP, pancreatic stump was closed by using a stapler transpancreatic mattress suture [16] based on the surgeons' preferences. In PD, either pancreatojejunostomy or pancreaticogastrostomy was performed based on the surgeons' preferences. In PD, pancreatojejunostomy was performed using 3–0 monofilaments, following duct‐to‐mucosa anastomosis using 5–0 monofilaments as we previously described [17]. Pancreaticogastrostomy was performed using 3–0 monofilaments. In either procedure, a pancreatic stent tube was placed to drain pancreatic juice externally.

3 Postoperative management

All the patients underwent our postoperative management protocol as previously described [17]. Briefly, in PD cases, surgical drains were placed under the hepatoduodenal ligament and ventral and dorsal sides of PJ anastomosis, whereas in DP cases, they were placed at pancreatic stump and in the left subphrenic space in cases with splenectomy and only at the stump in cases without splenectomy. Octreotide was administered when the drain amylase level exceeded 5000 U/L. In patients experiencing CR‐POPF, surgical drains were changed every 1–2 weeks until disappearance of intra‐abdominal cavities was confirmed radiologically.

4 K‐means analysis and predictive value for CR‐POPF

We clustered the patients undergoing DP into three clusters by the use of k‐means analysis based on three indices: BMI, pancreatic thickness, and CCI score. Next, in accordance with a previous report [18], an ordinal logistic regression model was used to estimate the weight (*W*) of each index in the cluster (*W*_BMI_, *W*_pancreatic thickness_, and *W*_CCI_). The predictive value was calculated by using these *W* values as coefficients:

$$\text{Predictive}\;\text{value}=W_{\text{BMI}}\times \text{BMI}\;\left(\text{kg}/m^{2}\right)+W_{\text{pancreas}\;\text{thickness}}\times \text{pancreas}\;\text{thickness}\;\left(\text{mm}\right)+W_{\text{CCI}}\times \text{CCI}$$

The predictive value of all the patients was obtained by the above formula, and the optimal cutoff value in relation to the occurrence of CR‐POPF was calculated by depicting a receiver operating characteristic (ROC) curve.

5 Statistical analysis

Patient characteristics were evaluated in association with the formation of CR‐POPF using the chi‐squared statistics and the Mann–Whitney *U* test. To determine the optimal cutoff levels of each continuous variable for predicting CR‐POPF, ROC curves (Youden index) were constructed in univariate and multivariate analyses. Variables with *p* < 0.05 were incorporated into a logistic regression model to determine independent risk factors for CR‐POPF, which are described with odds ratios. Statistical significance was defined as *p* < 0.05. All statistical analyses were conducted using the JMP software (JMP, version 13.2.1).

## 3 Results

1. Overall patients

The rate of CR‐POPF in patients in the overall cohort (*n* = 597) according to the CCI score was assessed. As summarized in Supporting Information Figure S1, the rate of CR‐POPF increased as the CCI score became higher. Next, we stratified these patients based on the surgical procedure (i.e., DP vs. PD), and their characteristics were summarized in Supporting Information Table S1. As shown in Table 1, CCI score had a significant association with the occurrence of CR‐POPF in the DP group (*p* = 0.0031), whereas it did not in the PD group (*p* > 0.9999).

2 Impact of CCI on POPF in the DP group

**TABLE 1:** The association between CCI score and CR‐POPF after DP and PD.

CCI score	DP			PD		
	CR‐POPF (+)	CR‐POPF (−)	*p* value	CR‐POPF (+)	CR‐POPF (−)	*p* value
CCI ≤ 4	35	163		109	233	
CCI > 4	10	11	0.0031	11	25	> 0.9999

Abbreviations: CCI, Charlson Comorbidity Index; CR‐POPF, clinically relevant pancreatic fistula; DP, distal pancreatectomy; PD, pancreaticoduodenectomy.

**FIGURE 2:**
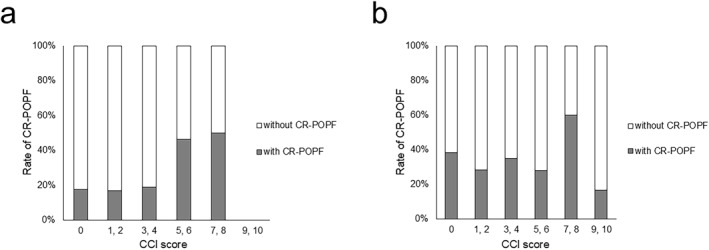
The association between Charlson Comorbidity Index score and the occurrence of CR‐POPF. The association between CCI score and the occurrence of CR‐POPF was summarized in the patients undergoing DP (a) and patients undergoing PD (b). (a) In patients undergoing DP, the occurrence of CR‐POPF increased in those with CCI score > 4 points compared with those with CCI score ≤ 4 points. (b) In patients undergoing PD, the rate of CR‐POPF was generally consistent regardless of CCI score. CCI, Charlson Comorbidity Index; CR‐POPF, clinically relevant postoperative pancreatic fistula; DP, distal pancreatectomy; PD, pancreatoduodenectomy.

First, we reviewed the DP group (*n* = 219). Figure 2a displays the rate of CR‐POPF according to CCI score in the DP group. An ROC curve was drawn to reveal that the optimal cutoff of CCI score in the DP group was four points. The rate of CR‐POPF significantly increased in the patients with CCI score > 4 points compared with those with CCI score ≤ 4 points (47.6% (10/21) versus 17.7% (35/198), *p* = 0.0031; Table 1). Therefore, the DP group was divided into two groups: a high‐risk group including patients with CCI score > 4 points (*n* = 21) and a low‐risk group including patients with CCI score ≤ 4 points (*n* = 198). The patients' characteristics were compared between the two groups (Table 2). The patients in the high‐risk group included significantly more patients who were male, experienced neoadjuvant therapy, underwent a longer operation, and experienced CR‐POPF than those in the low‐risk group. Table 3 summarizes the results of univariate and multivariate analyses to assess the factors associated with the occurrence of CR‐POPF. In the DP group, three factors were independently associated with CR‐POPF: BMI > 25.95 kg/m^2^, pancreatic thickness > 9.01 mm, and CCI score > 4 points (*p* = 0.0032, 0.0441, and 0.0364, respectively; Table 3).

3 Impact of CCI on POPF in the PD group

**TABLE 2:** Patients' Characteristics in the DP patients.

	High‐risk group (CCI score > 4; *n* = 21)	Low‐risk group (CCI score ≤ 4; *n* = 198)	*p* value
Preoperative factors			
Sex (male)	17 (80.95%)	104 (52.53%)	0.0192
Age (years old) (median, IQR)	69 (53–79)	69.5 (59–76)	0.5921
BMI (kg/m^2^) (median, IQR)	21.82 (20.18–23.53)	21.78 (20.03–24.15)	0.9701
NLR (median, IQR)	2.70 (1.82–3.75)	2.21 (1.64–3.25)	0.4591
Neoadjuvant therapy	1 (4.76%)	54 (27.27%)	0.0308
Pancreatic thickness (mm) (median, IQR)	10.53 (9.21–12.41)	10.22 (8.18–12.45)	0.6401
Histology (PDAC)	9 (42.86%)	90 (45.45%)	> 0.9999
Intraoperative factors			
Operative approach (laparotomy)	5 (23.81%)	60 (30.30%)	0.6233
Operative time (min) (median, IQR)	363 (256–431)	275 (224–334)	0.0079
Blood loss (mL) (median, IQR)	250 (125–720)	200 (70–443)	0.3107
Intraoperative transfusion	2 (9.52%)	16 (8.08%)	0.6855
Postoperative factor			
CR‐POPF	10 (47.62%)	35 (17.68%)	0.0031

Abbreviations: BMI, body mass index; CCI, Charlson Comorbidity Index; CR‐POPF, clinically relevant pancreatic fistula; DP, distal pancreatectomy; IQR, interquartile range; NLR, neutrophil‐lymphocyte ratio; PDAC, pancreatic ductal carcinoma.

**TABLE 3:** Univariate and multivariate analyses for CR‐POPF in DP patients.

	Univariate analysis				Multivariate analysis		
	*n*	OR	95% CI	*p* value	OR	95% CI	*p* value
Preoperative factors							
Sex (male vs. female)	31/14	2.0667	1.0287–4.1520	0.0443	1.9271	0.8648–4.2943	0.1086
Age (> 68 vs. ≤ 68 years old)	18/27	0.5292	0.2716–1.0313	0.0668			
BMI (> 25.95 vs. ≤ 25.95 kg/m^2^)	11/33	4.9091	1.9647–12.2659	0.0009	4.4731	1.6534–12.1014	0.0032
NLR (> 2.18 vs. ≤ 2.18)	30/15	2.0952	1.0529–4.1695	0.0437	1.7816	0.8359–3.7975	0.1347
Neoadjuvant therapy (yes vs. no)	7/38	0.4836	0.2022–1.1565	0.1230			
Pancreatic thickness (> 9.01 vs. ≤ 9.01 mm)	39/6	3.7818	1.5176–9.4241	0.0022	2.6844	1.0265–7.0196	0.0441
Histology (PDAC vs. others)	18/27	0.7654	0.3930–1.4908	0.5026			
CCI score (> 4 vs. ≤ 4 points)	14/31	2.3548	1.1129–4.9829	0.0325	3.024	1.0726–8.5260	0.0364
Intraoperative factors							
Operative approach (laparotomy vs. others)	33/12	0.8302	0.3979–1.7320	0.7157			
Operative time (> 278 vs. ≤ 278 min)	29/16	2.0335	1.0312–4.0103	0.0451	1.5348	0.7071–3.3316	0.2786
Blood loss (> 770 vs. ≤ 770 mL)	10/35	3.0286	1.2564–7.3006	0.0168	1.6692	0.6112–4.5586	0.3175
Intraoperative transfusion (yes vs. no)	5/40	1.5481	0.5215–4.5951	0.5410			

Abbreviations: BMI, body mass index; CCI, Charlson Comorbidity Index; CI, confidence interval; CR‐POPF, clinically relevant postoperative pancreatic fistula; DP, distal pancreatectomy; NLR, neutrophil‐lymphocyte ratio; OR, odds ratio; PDAC, pancreatic ductal carcinoma.

Then, we assessed the association between CCI score and CR‐POPF in the PD group (*n* = 378). As shown in Figure 2b, the rate of CR‐POPF was generally consistent regardless of CCI score without any significant difference. In the PD group, univariate and multivariate analyses revealed four independent variables that were associated with CR‐POPF: male sex, BMI > 21.21 kg/m^2^, pancreatic thickness > 12.80 mm, PDAC histology (*p* = 0.0038, 0.0004, 0.0325, and < 0.0001, respectively; Table 4).

4 K‐means cluster analysis and predictive value for CR‐POPF in the DP group

**TABLE 4:** Univariate and multivariate analyses for CR‐POPF in PD patients.

	Univariate analysis				Multivariate analysis		
	*n*	OR	95% CI	*p* value	OR	95% CI	*p* value
Preoperative factors							
Sex (male vs. female)	92/28	2.4811	1.5296–4.0482	0.0002	2.187	1.2865–3.7178	0.0038
Age (> 68 vs. ≤ 68 years old)	70/50	1.1986	0.7737–1.8568	0.4382			
BMI (> 21.21 vs. ≤ 21.21 kg/m^2^)	86/33	3.1184	1.9481–4.9917	< 0.0001	2.5327	1.5197–4.2210	0.0004
NLR (> 2.48 vs. ≤ 2.48)	43/76	0.6072	0.3881–0.9498	0.0337	0.7724	0.4708–1.2671	0.3065
Neoadjuvant therapy (yes vs. no)	18/102	0.3926	0.2229–0.6917	0.0010	0.7169	0.3236–1.5881	0.4121
Pancreatic thickness (> 12.80 vs. ≤ 12.80 mm)	69/51	2.2456	1.4448–3.4902	0.0004	1.7027	1.0454–2.7732	0.0325
Histology (PDAC vs. others)	19/101	0.2376	0.1373–0.4111	< 0.0001	0.2107	0.0995–0.4464	< 0.0001
CCI score (> 4 vs. ≤ 4 points)	11/109	0.9406	0.4466–1.9807	> 0.9999			
Intraoperative factors							
Operative approach (laparotomy vs. others)	118/2	0.7119	0.1416–3.5798	> 0.9999			
Operative time (> 410 vs. ≤ 410 min)	108/12	1.8505	0.9385–3.6487	0.0869			
Blood loss (> 1820 vs. ≤ 1820 mL)	7/113	0.6645	0.2757–1.6014	0.4130			
Intraoperative transfusion (yes vs. no)	28/92	0.8337	0.5032–1.3812	0.5282			

Abbreviations: BMI, body mass index; CCI, Charlson Comorbidity Index; CI, confidence interval; CR‐POPF, clinically relevant postoperative pancreatic fistula; NLR, neutrophil‐lymphocyte ratio; OR, odds ratio; PD, pancreatoduodenectomy; PDAC, pancreatic ductal carcinoma.

**FIGURE 3:**
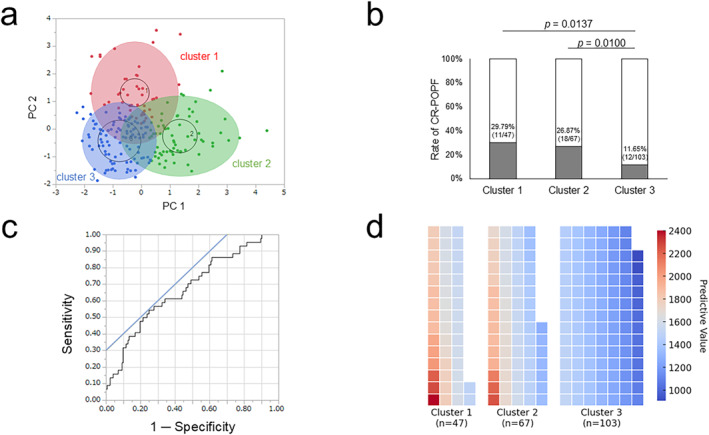
The occurrence and risk of CR‐POPF in the three clusters. Patients undergoing DP were classified into three clusters using *k*‐means analysis based on the three variables: BMI, pancreatic thickness, and CCI score. (a) In the cluster 3, the occurrence of CR‐POPF was significantly decreased compared with the other 2 clusters (11.65% vs. 29.79% and 26.87%; *p* = 0.0100 and 0.0137). (b) K‐means cluster analysis classified patients undergoing distal pancreatectomy into three clusters using three variables: BMI, pancreatic thickness, and CCI score. The red, green, and blue areas indicate the clusters 1, 2 and 3, respectively. (c) The association between the predictive value and the occurrence of CR‐POPF in the DP group was depicted in the ROC curve (AUC = 0.6750 [95% CI: 0.5804–0.7696]). The optimal cutoff value was 1573 to separate the low‐risk cohort from the high‐risk cohort. (d) A heatmap was drawn to represent the risk of CR‐POPF across the three clusters. Each square represents an individual patient, with colors indicating the predictive value of CR‐POPF risk, where red signifies higher risk and blue indicates lower risk. AUC, area under curve; BMI, body mass index; CCI, Charlson Comorbidity Score; CI, confidence interval; CR‐POPF, clinically relevant postoperative pancreatic fistula; DP, distal pancreatectomy; PC, principal component; ROC, receiver operating characteristic. *p < 0.05.

As discussed above, in the DP group, high BMI, thick pancreas, and high CCI score were significantly associated with an increased occurrence of CR‐POPF. We divided the DP group into three clusters by using k‐means cluster analysis based on these three variables. The biplot of *k*‐means analysis was shown in Figure 3a. The occurrence of CR‐POPF was significantly lower in the cluster 3 compared with the clusters 1 and 2 (*p* = 0.0137 and = 0.0100; Figure 3b). Based on this clustering, the predictive value for the occurrence of CR‐POPF was calculated as follows:

$$\text{Predictive}\;\text{value}=34\times \text{BMI}\;\left(\text{kg}/m^{2}\right)+41\times \text{pancreas}\;\text{thickness}\;\left(\text{mm}\right)+91\times \text{CCI}$$

The predictive value was obtained from all the DP group using this formula, and an ROC curve was depicted to calculate the optimal cutoff of the predictive value in association with the development of CR‐POPF (Figure 3c; area under the curve = 0.6750 [95% confidence interval: 0.5804–0.7696]). As a result, patients with the predictive value > 1573 were considered high‐risk for CR‐POPF (35.82% (24/67)) and low‐risk (13.33% (20/150)) if otherwise. The accuracy of this risk stratification was 70.97% with sensitivity being 54.55%, specificity being 75.14%, and negative predictive value being 86.67% (Table 5). Additionally, a heatmap was generated to visualize the risk of CR‐POPF across the three clusters (Figure 3d). Each square represents an individual patient, with colors indicating the predictive value of CR‐POPF risk, where red signifies higher risk and blue represents lower risk. The risk of CR‐POPF was highest in Cluster 1 and progressively decreased in Clusters 2 and 3. Finally, the clinical impact of the predictive formula was verified in the validation cohort. The patient characteristics of the validation cohort were summarized in Supporting Information Table S2. As shown in the Supporting Information Table S3, the predictive formula yielded a high negative predictive value (86.36%), although in the validation cohort, the significant difference was not observed with regard to the occurrence of CR‐POPF (*p* = 0.6527).

**TABLE 5:** Risk based on the predictive value and the occurrence of CR‐POPF.

	CR‐POPF	
	Yes	No
Low risk (predictive value ≤ 1573; *n* = 150)	20 (13.33%)	130 (86.67%)
High risk (predictive value > 1573; *n* = 67)	24 (35.82%)	43 (64.18%)

Abbreviation: CR‐POPF, clinically relevant postoperative pancreatic fistula.

## 4 Discussion

CCI was originally invented to predict the inpatients' prognosis [9]. However, recent reports describe a significant association between CCI and postoperative complications in several surgical fields [12, 13]. Wada et al. described that the presence of severe comorbidities is associated with a low peripheral blood supply after right‐sided colon cancer resection, which may give rise to an increased probability of postoperative complications [12]. The formation of POPF carries multifactorial pathogenesis. All of the preoperative, intraoperative, and postoperative factors collaboratively act on its development [19, 20, 21]. Despite the unmodifiable nature of preoperative factors (e.g., sex, age, and comorbidities), it is still valuable to preoperatively assess the risk of POPF because high‐risk patients may warrant preoperative nutritional support [2, 22] or postoperative use of somatostatin analogs [3], whereas low‐risk patients may benefit from more aggressive postoperative management such as the early drain removal strategy [4]. In this context, in the current study, the impact of comorbidities on the formation of POPF was evaluated by utilizing CCI as a scoring system to show the comprehensive severity of a patient's comorbidities.

Our results showed that CCI score was an independent factor significantly associated with the development of CR‐POPF in patients undergoing DP (*p* = 0.0364, Table 3). Although the reason for the higher frequency of CR‐POPF in patients with more severe comorbidities has never been explained before, pancreatic steatosis may be one of the reasons that explains it. Fukuda et al. reported the impact of pancreatic steatosis as one of the potential determinants for the formation of POPF [23], which is also reported to be associated with multiple comorbidities such as diabetes mellitus [24], hyperlipidemia [25], and fatty liver [25]. That is, severe comorbidities cause the histological change in the pancreas, which may give rise to POPF. Interestingly, none of the individual item composing CCI had a significant association with CR‐POPF in this population (Supporting Information Table S4). These results suggest that it is comprehensive severity of comorbidities rather than a specific comorbidity that has an impact of the formation of CR‐POPF. Previous reports show that some of the comorbidities, such as diabetes mellitus [7] or kidney dysfunction [8], are associated with POPF. However, even in the presence or absence of these specific comorbidities, patients are at a risk of developing CR‐POPF if CCI is more than four points.

On the contrary, in the patients undergoing PD, CCI score was not significantly associated with the formation of CR‐POPF (*p* > 0.9999; Table 4). The reason for this result may be explained by the technical demand of pancreatojejunostomy in PD. That is, the surgical complexity of pancreatojejunostomy in PD makes the pancreatic status more significantly associated with POPF, whereas comparatively simple stump closure procedure in DP provides more room for the severity of comorbidities to have an impact on the formation of POPF. Moreover, the insignificant impact of CCI on CR‐POPF in the PD group may partly be explained by an effect modification of pancreatic thickness. Specifically, when stratified by the mean pancreatic thickness (12.08 mm), a CCI > 4 points was associated with a lower rate of CR‐POPF in patients with a thick pancreas (> 12.08 mm) (odds ratio: 0.6030), but a higher rate of CR‐POPF in those with a thin pancreas (≤ 12.08 mm) (odds ratio: 1.5714) (Supporting Information Table S5). In our patient cohort, the pancreas was significantly thicker in the PD group compared to the DP group (*p* < 0.0001; Supporting Information Table S1). Given that a thick pancreas is associated with a lower risk of CR‐POPF when CCI is high, the predominance of thick pancreas in the PD group may have mitigated the overall effect of CCI on CR‐POPF, leading to an insignificant association in this population.

Previous reports describe the usefulness of scoring systems to predict POPF [26, 27]. However, these reports do not include CCI scores in their scoring system, although the current study demonstrated that patients' comorbidities need to be considered in assessing the risk for POPF. In this context, we attempted to devise a predictive formula to include CCI as one of the components of the formula. First, we classified our patients into three clusters using k‐means analysis. *K*‐means analysis is easy to perform using a basic statistical software, making it widely accessible to a broad population. As a result, the cluster 3 was identified as the significantly low‐risk cohort for CR‐POPF (11.65%) compared with the other two clusters (Figure 3b). Next, we devised a formula to calculate the predictive score for the occurrence of CR‐POPF. As the formula shows, CCI has a greater impact on the predictive value than BMI and pancreatic thickness do. Moreover, this formula is beneficial to identify low‐risk patients for CR‐POPF (i.e., patients with predictive value ≤ 1573) considering the low negative predictive value of this formula (86.67%; Table 5). External validation was performed enrolling patients undergoing DP from 2021 to 2023 at our institution. As a result, our predictive model yielded as high as 86.36% negative predictive value, reinforcing its ability to identify patients at low risk for CR‐POPF post‐DP, despite the absence of a statistically significant association with CR‐POPF (*p* = 0.6527 and Supporting Information Table S3). Although a high positive predictive value is desirable in predictive models of this kind (e.g., fistula risk score [28]), a high negative predictive value remains valuable as it may contribute to a reduction in prolonged antibiotic use, unnecessary surgical drain placement, and ultimately, a shorter length of hospital stay.

There are limitations to be addressed in this study. First, since its retrospective nature, we must be cautious in interpreting the results of this study. Specifically, patients with severe comorbidities might have been considered ineligible for PD, which may have led to a selection bias in the study. Second, the occurrence of POPF varies according to the patients' background. For example, patients with pancreatic cancer are less likely to develop POPF than those with benign pancreatic lesions. Thus, predictive formula may be alternatively devised in assessing the rate of POPF in other cohorts with different patient backgrounds. Third, since this study exclusively enrolled Japanese patients, BMI values are relatively low; therefore, the findings may not be directly applicable to the Western population. Finally, future studies are warranted to explore more accurate predictive formulas considering the moderate accuracy (AUC = 0.6750) of our predictive model.

In conclusion, CCI was a significant predictive factor for POPF after DP, whereas it was not associated with CR‐POPF after PD. Patients with CCI > 4 points may warrant prudent postoperative management after DP. The combination of CCI with other clinical factors may provide a predictive model to identify low‐risk population for CR‐POPF.

## Author Contributions

**Hiroki Imamura:** conceptualization, data curation, formal analysis, investigation, methodology, project administration, resources, writing – original draft, writing – review and editing. **Yoshito Tomimaru:** conceptualization, data curation, formal analysis, investigation, methodology, project administration, resources, supervision, visualization, writing – review and editing. **Shogo Kobayashi:** conceptualization, formal analysis, investigation, methodology, project administration, resources, supervision, visualization, writing – review and editing. **Kazuki Sasaki:** conceptualization, formal analysis, investigation, methodology, project administration, resources, supervision, visualization, writing – review and editing. **Shinichiro Hasegawa:** conceptualization, data curation, formal analysis, investigation, methodology, project administration, resources, supervision, validation, visualization, writing – review and editing. **Daisaku Yamada:** conceptualization, formal analysis, investigation, methodology, project administration, resources, supervision, visualization, writing – review and editing. **Hirofumi Akita:** conceptualization, data curation, formal analysis, investigation, methodology, project administration, resources, supervision, validation, visualization, writing – review and editing. **Takehiro Noda:** conceptualization, formal analysis, investigation, methodology, project administration, resources, supervision, visualization, writing – review and editing. **Hidenori Takahashi:** conceptualization, formal analysis, investigation, methodology, project administration, resources, supervision, visualization, writing – review and editing. **Yuichiro Doki:** conceptualization, formal analysis, investigation, methodology, project administration, resources, supervision, visualization, writing – review and editing. **Hidetoshi Eguchi:** conceptualization, formal analysis, investigation, methodology, project administration, resources, supervision, visualization, writing – review and editing.

## Ethics Statement

The current study was performed in compliance with the Declaration of Helsinki and was approved by the ethical committee of our hospital (approval number 24079).

## Consent

Informed consent was obtained from all individual participants included in the study.

## Conflicts of Interest

The authors declare no conflicts of interest.

5

## Supporting information

Supporting Information S1

## Data Availability

The data that support the findings of this study are available from the corresponding author upon reasonable request.
