# Supplementary material for: The Charlson Comorbidity Index Predicts Clinically Relevant Postoperative Pancreatic Fistula in Patients Undergoing Distal Pancreatectomy Not Pancreaticoduodenectomy
Source: World J Surg. 2025 Mar 24;49(5):1298–305. doi: 10.1002/wjs.12557 (PMC12058436; doi:10.1002/wjs.12557)
Supplement: Supplementary file 1 — Supporting Information S1 [file WJS-49-1298-s001.docx]

Supplementary Material

Article Title:

Charlson Comorbidity Index Predicts Clinically Relevant Postoperative Pancreatic Fistula in Patients Undergoing Distal Pancreatectomy Not Pancreaticoduodenectomy

Journal Name:

World Journal of Surgery

Author Names:

Hiroki Imamura, Yoshito Tomimaru, Shogo Kobayashi, Kazuki Sasaki, Shinichiro Hasegawa, Daisaku Yamada, Hirofumi Akita, Takehiro Noda, Hidenori Takahashi, Yuichiro Doki, Hidetoshi Eguchi

Corresponding Author:

Shogo Kobayashi, MD, PhD, FACS

Department of Gastroenterological Surgery, Graduate School of Medicine, Osaka University

2-2 Yamada-oka, Suita, Osaka 565-0871, Japan

Tel: +81-6-6879-3251, Fax: +81- 6-6879-3259

Email: [skobayashi@gesurg.med.osaka-u.ac.jp](mailto:skobayashi@gesurg.med.osaka-u.ac.jp)

| Supplementary Table 1 Patients' Characteristics in the Overall Cohort | | |  |  |  |  |
| --- | --- | --- | --- | --- | --- | --- |
|  |  | PD patients (n=378) |  | DP patients (n=219) |  | p value |
| ***Preoperative factors*** | |  |  |  |  |  |
|  | Sex (male) | 239 (63.23%) |  | 121 (55.25%) |  | 0.0568 |
|  | Age (years old) (median, IQR) | 70 (62–75) |  | 69 (59–76) |  | 0.0023 |
|  | BMI (kg/m^2^) (median, IQR) | 21.57 (19.31–23.84) |  | 21.82 (20.08–24.12) |  | 0.1509 |
|  | NLR (median, IQR) | 2.31 (1.70–3.29) |  | 2.23 (1.65–3.27) |  | 0.2211 |
|  | Neoadjuvant therapy | 98 (25.93%) |  | 55 (25.11%) |  | 0.8464 |
|  | Pancreatic thickness (mm) (median, IQR) | 12.08 (9.97–14.21) |  | 10.27 (8.45–12.44) |  | <0.0001 |
|  | Histology (PDAC) | 133 (35.19%) |  | 99 (45.21%) |  | 0.0186 |
|  | CCI score (median, IQR) | 2 (2–3) |  | 2 (0–3) |  | 0.0034 |
| ***Intraoperative factors*** | |  |  |  |  |  |
|  | Operative approach (laparotomy) | 8 (2.12%) |  | 65 (29.68%) |  | <0.0001 |
|  | Operative time (min) (median, IQR) | 517 (445.75–597.25) |  | 279 (225–344) |  | <0.0001 |
|  | Blood loss (mL) (median, IQR) | 620 (380–1020) |  | 200 (70–450) |  | <0.0001 |
|  | Intraoperative transfusion | 97 (25.66%) |  | 18 (8.22%) |  | <0.0001 |
| ***Postoperative factor*** | |  |  |  |  |  |
|  | CR-POPF | 120 (31.75%) |  | 45 (20.55%) |  | 0.0032 |
| PD, pancreatoduodenectomy; DP, distal pancreatectomy; IQR, interquartile range; BMI, body mass index; NLR, neutrophil-lymphocyte ratio; PDAC, pancreatic ductal carcinoma; CCI, Charlson Comorbidity Index; CR-POPF, clinically relevant pancreatic fistula | | | | | | |

| Supplementary Table 2 Patients' Characteristics in the Validation Cohort | | |
| --- | --- | --- |
|  |  | n=77 |
| ***Preoperative factors*** | |  |
|  | Sex (male) | 37 (48.05%) |
|  | Age (years old) (median, IQR) | 71 (55–77) |
|  | BMI (kg/m^2^) (median, IQR) | 22.62 (20.18–24.70) |
|  | NLR (median, IQR) | 2.57 (1.73–3.35) |
|  | Neoadjuvant therapy | 34 (44.16%) |
|  | Pancreatic thickness (mm) (median, IQR) | 8.84 (7.44–10.54) |
|  | Histology (PDAC) | 41 (53.25%) |
|  | CCI score (median, IQR) | 1 (0–2) |
| ***Intraoperative factors*** | |  |
|  | Operative approach (laparotomy) | 21 (27.27%) |
|  | Operative time (min) (median, IQR) | 342 (271–405) |
|  | Blood loss (mL) (median, IQR) | 130 (35–290) |
|  | Intraoperative transfusion | 4 (5.20%) |
| ***Postoperative factor*** | |  |
|  | CR-POPF | 11 (14.29%) |
| IQR, interquartile range; BMI, body mass index; NLR, neutrophil-lymphocyte ratio; PDAC, pancreatic ductal carcinoma; CCI, Charlson Comorbidity Index; CR-POPF, clinically relevant pancreatic fistula | | |

| Supplementary Table 3 Risk Based on the Predictive Value and the Occurrence of CR-POPF in the Validation Cohort | | | | | |
| --- | --- | --- | --- | --- | --- |
|  |  | CR-POPF | |  |  |
|  |  | yes | no |  |  |
| Low risk (predictive value ≤ 1573; n=66) |  | 9 (13.64%) | 57 (86.36%) |  |  |
| High risk (predictive value > 1573; n=11) |  | 2 (18.18%) | 9 (81.82%) |  |  |
| CR-POPF, clinically relevant postoperative pancreatic fistula | | |  |  |  |

Supplementary Table 4 Items of Charlson Comorbidity Index in Association with CR-POPF in DP Patients

| Comorbidity | n |  | CR-POPF |  | p value |
| --- | --- | --- | --- | --- | --- |
| Myocardial infarction | 0 |  | 0 |  | N/A |
| Congestive heart failure | 2 |  | 0 (0%) |  | >0.9999 |
| Peripheral vascular disease | 1 |  | 1 (100%) |  | 0.2055 |
| Cerebrovascular disease | 7 |  | 2 (28.57%) |  | 0.6346 |
| Dementia | 0 |  | 0 |  | N/A |
| Chronic pulmonary disease | 3 |  | 0 (0%) |  | >0.9999 |
| Connective tissue disease | 0 |  | 0 |  | N/A |
| Peptic ulcer disease | 49 |  | 8 (16.33%) |  | 0.5473 |
| Mild liver disease | 5 |  | 2 (40%) |  | 0.2731 |
| Diabetes without end-organ damage | 30 |  | 5 (16.67%) |  | 0.8078 |
| Hemiplegia | 0 |  | 0 |  | N/A |
| Moderate or severe renal disease | 4 |  | 2 (50%) |  | 0.1879 |
| Diabetes with end-organ damage | 21 |  | 7 (33.33%) |  | 0.1539 |
| Tumor without metastasis | 132 |  | 30 (22.73%) |  | 0.3938 |
| Leukemia | 1 |  | 0 (0%) |  | >0.9999 |
| Lymphoma | 1 |  | 1 (100%) |  | 0.2055 |
| Moderate or severe liver disease | 0 |  | 0 |  | N/A |
| Metastatic solid tumor | 11 |  | 5 (45.45%) |  | 0.0512 |
| AIDS | 0 |  | 0 |  | N/A |

CR-POPF, clinically relevant postoperative pancreatic fistula; DP, distal pancreatectomy;

AIDS, acquired immunodeficiency syndrome

Supplementary Table 5 The Association between CCI score and CR-POPF in PD patients

| Pancreatic thickness (mm) | CCI score |  | CR-POPF | OR | p value |
| --- | --- | --- | --- | --- | --- |
| > 12.08 | ≤4 |  | 67 (39.88%) |  |  |
|  | >4 |  | 6 (28.57%) | 0.6030 | 0.3527 |
|  |  |  |  |  |  |
| ≤ 12.08 | ≤4 |  | 42 (24.14%) |  |  |
|  | >4 |  | 5 (33.33%) | 1.5714 | 0.5329 |

CCI, Charlson Comorbidity Index; CR-POPF, clinically relevant postoperative pancreatic fistula; PD, pancreatoduodenectomy; OR, odds ratio

Supplementary Fig. 1


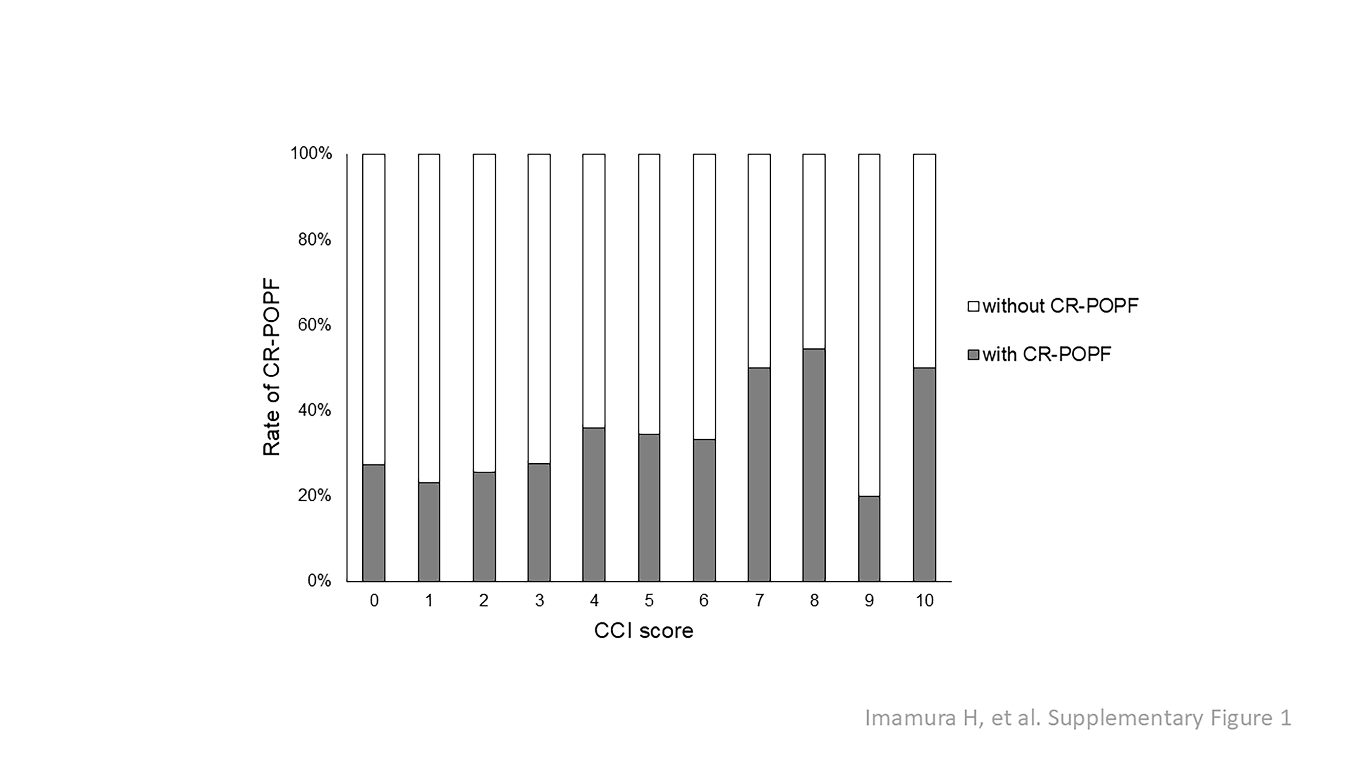


Supplementary Fig.1 The Association between Charlson Comorbidity Index Score and the Occurrence of CR-POPF in the overall cohort

The association between CCI score and the occurrence of CR-POPF was summarized in the overall patients. In the overall cohort, the occurrence of CR-POPF gradually increased as CCI score became higher.
